# Influence of commercial inactivated yeast derivatives on the survival of probiotic bacterium *Lactobacillus rhamnosus* HN001 in an acidic environment

**DOI:** 10.1186/s13568-017-0456-4

**Published:** 2017-07-24

**Authors:** Mingzhan Toh, Shao Quan Liu

**Affiliations:** 1 Food Science and Technology Programme, Department of Chemistry, National University of Singapore, 3 Science Drive 3, Singapore, 117543 Singapore; 2 National University of Singapore (Suzhou) Research Institute, No. 377 Linquan Street, Suzhou Industrial Park, Suzhou, 215123 Jiangsu China

**Keywords:** *Lactobacillus rhamnosus*, Inactivated yeast derivatives, Acid stress, Viability, Probiotics

## Abstract

This study evaluated the influence of three inactivated yeast derivatives (IYDs) used in wine production, namely OptiRed^®^, OptiWhite^®^ and Noblesse^®^, on the viability of the probiotic strain *Lactobacillus rhamnosus* HN001 in an acidic environment. Addition of the IYDs at 3 g/L significantly enhanced the survival of the probiotic bacteria by 2.75–4.05 log cycles after 10-h exposure in a pH 3.0 buffer. Acid stress assay with IYD components obtained after centrifugation and filtration revealed that water-soluble compounds were responsible for improving the acid tolerance of *L. rhamnosus* HN001 for all three preparations. Differences in protective effect amongst the IYDs on *L. rhamnosus* HN001 were observed when permeates and retentates of the water-soluble extracts, obtained through ultrafiltration with a 2 kDa membrane, were assayed against the lactic acid bacterium. Chemical analysis of the water-soluble components suggests that low molecular weight polysaccharides, specific free amino acids and/or antioxidants in the 2 kDa permeates could have contributed to the enhanced survival of *L. rhamnosus* HN001 during acid stress. The contrast amongst the 2 kDa retentates’ viability enhancing property may have been attributed to the differences in size and structure of the higher molecular weight carbohydrates and proteins, as the survival of the probiotic did not relate to the concentration of these compounds. These results suggests that oenological IYDs could potentially be applied to probiotic foods for enhancing the acid tolerance of the beneficial microorganisms, and consequently prolonging the shelf life of these products.

**Electronic supplementary material:**

The online version of this article (doi:10.1186/s13568-017-0456-4) contains supplementary material, which is available to authorized users.

## Introduction

Enhancing the survival of probiotic bacteria in acidic environments such as fermented food carriers and gastric juice is crucial for ensuring their optimal function as health promoting agents. As defined by the FAO/WHO working group, probiotics are “live microorganisms that, when administered in adequate amounts, confer a health benefit on the host” (FAO/WHO 2001). It is generally accepted that the minimum level of these microorganisms in probiotic foods or supplements at the point of consumption is 1 × 10^9^ CFU per serving, or 1 × 10^7^ CFU/g, for delivering sufficient viable cells required to colonize the host’s gut (Hill et al. 2014).

Dairy products like yogurt, kefir and cheese are the most popular vehicles for delivering probiotics to consumers. These food products are manufactured by fermentation with bacterial and/or yeast starter cultures, together with the probiotic bacteria, for improving the organoleptic properties of the milk and to allow propagation of the beneficial microorganisms to sufficient numbers required to exert their health effects (Heller 2001). During fermentation, lactic acid is produced in the cytoplasm of starter and probiotic lactic acid bacteria (LAB) as an end-product of glycolysis. The acid generated is excreted out of the bacteria cell via facilitated diffusion with lactate-proton symporters, causing a reduction in the milk’s pH (Gätje et al. 1991). Temperature fluctuation and abuse during distribution and storage of probiotic products can result in post-production acidification due to continuous generation of acidic metabolites by the bacteria (Liu and Tsao 2009). As the pH of the food matrix declines further, the proportion of organic acids existing in the non-dissociated form increases. Non-dissociated acids can diffuse passively across the cell membrane and dissociate in the more alkaline cytoplasm (Russell and Diez-Gonzalez 1997). The lowering of the intracellular pH and accumulation of anions disrupts metabolic processes crucial for the survival of the probiotic, leading to a reduction in viable cell counts and shortening the shelf life of the product (Wang et al. 2014).

Various approaches involving microencapsulation of probiotic cells, use of low acidifying, robust strains, co-culturing with other microorganisms and nutrient supplementation have been investigated to mitigate the effects of acidic stress on probiotics in food (Liu and Tsao 2009; Özer et al. 2005; Reid et al. 2007). Recent studies have also demonstrated improvements in probiotic acid tolerance brought about by the application of autoclaved whole yeast cells and yeast cell wall polysaccharides (Ganan et al. 2012; Lim et al. 2015; Rosburg et al. 2010). These polysaccharides are mainly β-glucans and mannans from mannoproteins, constituting approximately 60 and 40% of yeast cell wall dry mass, respectively (Aguilar et al. 2012). It was proposed that yeast parietal polysaccharides enhanced the viability of probiotics in acidic environments by providing carbon sources for energy production, as well as creating a physical barrier around the bacterial cells to shield them from the adverse environment (Russo et al. 2012; Stack et al. 2010). Apart from prolonging the survival of probiotics, β-glucans and mannoproteins have been reported to exert immunostimulatory, anti-inflammatory and antimicrobial effects by stimulating neutrophil and macrophage activities in the host (El Khoury et al. 2012; Ha et al. 2006; LeBlanc et al. 2006). Therefore, yeast polysaccharides could potentially be used as multifunctional agents in probiotic food products to improve their health benefits.

The use of yeast cell components, in the form of inactivated yeast derivatives (IYD), has been gaining traction in recent years in the oenological industry as additives to improve the fermentation and sensorial quality of wines (Pozo-Bayon et al. 2009). IYDs are produced by subjecting inactivated oenological yeast cells to enzymatic and/or physico-chemical treatments for obtaining specific cellular fractions. (Ángeles Pozo-Bayón et al. 2009). The composition of these preparations varies accordingly with their applications. For instance, IYDs with high quantities of mannoproteins are used in red wines for their ability to complex with polyphenols for color stabilization and reducing astringency, while glutathione-enriched IYDs are used in white wines to prevent enzymatic and oxidative browning (Escot et al. 2001; Kritzinger et al. 2013). IYDs can be added to nitrogen-deficient grape musts to provide assimilable amino nitrogen required for yeasts to carry out alcoholic fermentation (Ángeles Pozo-Bayón et al. 2009). They are also supplemented into wines during malolactic fermentation to compensate for the nitrogenous compounds utilized by the yeasts during alcoholic fermentation. The growth of wine LAB (*Oenococcus oeni, Lactobacillus hilgardii and Pediococcus pentosaceus*) involved in malolactic fermentation was stimulated by IYDs, which was attributed to the amino acids and monosaccharides provided by the preparations (Andujar-Ortiz et al. 2010).

Although IYDs have beneficial properties towards oenological LAB, their effect on probiotic LAB has yet to be explored. Therefore, the objective of this pilot study was to investigate the influence of three commercial IYDs that are rich in yeast parietal molecules on the survival of a widely used probiotic bacteria strain, *Lactobacillus rhamnosus* HN001, in a simulated acidic environment (buffer). As the IYDs studied are derived from *S. cerevisiae* of oenological origin, the findings of this study could also shed light on the survival-enhancing properties of non-viable *S. cerevisiae* EC-1118 cells on *L. rhamnosus* HN001 as previously reported by Suharja et al. (2014) and Lim et al. (2015).

## Materials and methods

### Microorganisms and culture conditions

The probiotic and yeast strain used in this study were *Lactobacillus rhamnosus* HN001 (Danisco A/S, Copenhagen, Denmark) and *Saccharomyces cerevisiae* EC-1118 (Lallemand Pty, Ontario, Canada), respectively.

*Lactobacillus rhamnosus* HN001 cells were grown by inoculating 1% (v/v) frozen stock culture into de Man, Rogosa, Sharpe (MRS) broth (Oxoid Ltd., Hampshire, England), followed by static incubation at 37 °C for 24 h. Yeast cells were propagated by inoculating 1% (v/v) frozen *S. cerevisiae* EC-1118 stock culture into yeast-malt (YM) broth (10 g/L dextrose (Sigma-Aldrich, Oakville, Ontario, Canada), 3 g/L yeast extract, 3 g/L malt extract and 5 g/L bacteriological peptone (all from Oxoid Ltd.), that was acidified to pH 5.0 with 1 M HCl and incubated statically at 30 °C for 24 h.

After two consecutive transfers, the microbial cultures were centrifuged (8000×*g*, 10 min, 4 °C) and washed twice with sterile 0.85% (w/v) NaCl. Working cultures were obtained by re-suspending the washed pellets to their initial volume with phosphate-buffered saline (PBS) that was acidified to pH 3.0 using 90% lactic acid (Merck, Darmstadt, Germany). All pH measurements were made using a Metrohm 713 pH meter (Herisau, Switzerland) calibrated with pH 4 and 7 buffers from Merck.

### Preparation of inactivated yeast derivative (IYD) extracts

Three inactivated yeast derivatives (IYDs) used in winemaking, OptiRed^®^, OptiWhite^®^ and Noblesse^®^, were purchased from Lallemand Pty. According to the manufacturer, OptiRed^®^ is used in red wines, OptiWhite^®^ for white and rose wines, while Noblesse^®^ could be used for all three types of wines. IYD extracts were prepared by suspending the IYD powders in PBS at 6 g/L, and the pH of the suspension was adjusted to 3.0 with lactic acid. The acidified IYD suspensions were sterilized at 121 °C for 15 min in an autoclave.

### Effect of IYDs on the survival of *L. rhamnosus* HN001 in an acidic buffer

An acid stress assay, modified from the method of Lim et al. (2015), was used to assess the impact of IYDs on the survival of *L. rhamnosus* HN001 in an acidic buffer. Twenty mL of *L. rhamnosus* HN001 working culture was mixed with an equal volume of IYD extract (giving a final IYD concentration of 3 g/L or 0.3% (w/v)) or pH 3.0 PBS for the control in a 50-mL polypropylene centrifuge tube and incubated statically in a 30 °C incubator for 10 h. As viable *S. cerevisiae* EC-1118 cells were demonstrated to enhance the viability of *L. rhamnosus* HN001 under similar stress conditions, an assay comprising of equal volumes probiotic bacteria and yeast working cultures was also performed for comparing the IYDs’ probiotic viability enhancing effect (Lim et al. 2015). An 1-mL aliquot was withdrawn every 2 h from the probiotic cell suspensions and serially diluted with 0.1% w/v buffered peptone water (Merck) for the enumeration of viable microbial counts. The viable *L. rhamnosus* HN001 count was determined using the pour plate method with MRS agar (Merck) supplemented with 0.1 g/L Natamax^®^ (50% Natamycin; Danisco A/S) followed by incubation at 37 °C for 48 h. Glucose-yeast extract agar containing 0.1 g/L oxytetracycline (Oxoid) was used for enumerating viable yeasts counts via the spread plate method followed by incubation at 30 °C for 48 h.

### Viability enhancing effects of water-soluble and insoluble fractions of IYD extracts

To investigate the components in IYDs that were responsible for enhancing the survival of *L. rhamnosus* HN001 in an acidic buffer, 20 mL of IYD extracts were centrifuged at 20,000×*g* for 30 min at 4 °C to obtain the water-soluble and insoluble fractions. The water-soluble fractions were prepared by filtering the supernatants of IYD extracts through sterile 0.20-μm regenerated cellulose (RC) syringe filters (Sartorius Stedium Biotech, Geottingen, Germany) and made up to 20 mL with pH 3.0 PBS. The pellets of IYD extracts were washed twice and made up to 20 mL with pH 3.0 PBS to obtain the water-insoluble fraction. Acid stress assays were performed as described above by mixing equal volumes of *L. rhamnosus* HN001 working culture with pH 3.0 PBS, whole, water-soluble or insoluble fractions of IYD extracts and incubating at 30 °C for 10 h. The log cycle reduction (Log (N_0h_/N_10h_)) of viable *L. rhamnosus* HN001 counts for each assay was determined by enumerating the probiotic counts before (N_0h_) and after (N_10h_) the incubation period.

### Ultrafiltration of water-soluble IYD fractions and viability enhancing effects of permeates and retentates

The water-soluble fractions of IYD extracts were further fractionated by ultrafiltration to characterize the nature of the viability-enhancing compounds present. Twelve mL of each water-soluble IYD fractions were loaded into a 2 kDa molecular weight cut off (MWCO) Hydrosart^®^ membrane Vivaspin 15R centrifugal concentrator (Sartorius Stedium Biotech) and centrifuged at 6000×*g* for 30 min at 4 °C. The obtained permeates and retentates were made up to 12 mL with pH 3.0 PBS and filter-sterilized with 0.20-μm RC syringe filters. The probiotic viability-enhancing effect of each permeate and retentate was assessed with the acid stress assay described above by combining 6 mL of *L. rhamnosus* HN001 working culture with an equal volume of the ultrafiltered fractions. The effect of permeates and retentates on the aggregation of *L. rhamnosus* HN001 was also examined according to the method of Lim et al. (2015) with modifications. Briefly, 3 mL of *L. rhamnosus* HN001 working culture was mixed with 3 mL of 2 kDa permeates, retentate or pH 3.0 PBS for the control. The cell suspensions were incubated at 30 °C and 0.2 mL of the suspension was withdrawn from the top fourth at 2-h intervals for 10 h. The optical density of withdrawn samples at 600 nm was measured with a microplate reader (Multiskan Spectrum, Thermo Scientific, Milford, MA, USA) and expressed as a proportion of the initial value.

### Chemical analyses of permeates and retentates of water-soluble IYD fractions

The free sugars (mono- and disaccharides) and amino acids content in permeates and retentates of the water-soluble IYD fractions after ultrafiltration were analysed with a Shimadzu Prominence ultra-fast liquid chromatography (UFLC) system (Kyoto, Japan). Chromatographic separation of free sugars was performed using a Zorbax carbohydrate column (150 × 4.6 mm. Agilent, Santa Clara, CA, USA) and 80% v/v acetonitrile with a flow rate of 1 mL/min at 30 °C. An evaporative light scattering detector (ELSD-LT II, Shimadzu) was used for the detection of eluted sugars. Free amino acids and ammonia were derivatized with 6-aminoquinolyl-*N*-hydroxysuccinimidyl carbamate (AQC) using the Waters AccQ-Tag Ultra Chemistry Kit (Dublin, Ireland), followed by chromatographic analysis according to the manufacturer’s protocol. The total free amino acid concentration was calculated by summing the amounts of ammonia and individual amino acids detected. Total carbohydrates were quantified using the phenol–sulfuric acid method as described by DuBois et al. (1956) and expressed as (mg glucose/mL). The total protein in each fraction was determined by the Bradford method using the Bio-Rad Protein Assay kit (Bio-Rad Laboratories, Hercules, California, USA), with bovine serum albumin (Sigma-Aldrich) as the standard. The total antioxidant capacity of the IYD ultrafiltered-fractions were measured using 1,1-diphenyl-2-picrylhydrazyl (DPPH) and ferric reducing ability of plasma (FRAP) assays according to the methods of Brand-Williams et al. (1995) and Amamcharla and Metzger (2014), respectively, and blanks were prepared by adding pH 3.0 PBS in place of the samples. Trolox (Sigma-Aldrich) was used as the standard for both antioxidant assays, and the DPPH radical scavenging activity and FRAP value of permeates and retentates were expressed in terms of Trolox equivalents (μg TE/mL).

### Statistical analysis

Mean values and standard deviations were calculated from data obtained from three independent experiments. All experimental data were analysed using one-way ANOVA and Duncan’s multiple range test with SPSS (Statistical Program for Social Sciences, SPSS Corporation, Chicago, IL) version 20.0 and differences were considered significant if *P* < 0.05.

## Results

### Probiotic viability enhancing effect of inactivated yeast derivatives in an acidic buffer

**Fig. 1:**
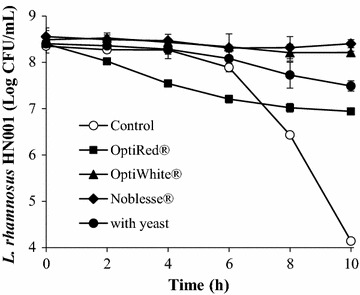
Effect of yeast and IYDs on the survival of *L. rhamnosus* HN001 at pH 3.0. Cell counts are the mean values of triplicate experiments (*n* = 3), with *error bars* representing the standard deviation of the mean values

The survival *of L. rhamnosus* HN001 in a pH 3 buffer was monitored bi-hourly over a 10-h duration at 30 °C (Fig. 1) in the absence and presence of 0.3% w/v IYDs or 7.56 Log CFU/mL viable *S. cerevisiae* EC-1118 cells. The pH of the assay medium after the 10-h incubation period was also measured, and no significant difference between the initial and terminal pH values was observed. For the negative control assay that was devoid of IYD and live yeast, the viability of *L. rhamnosus* HN001 declined sharply after 6 h, leading to a 4.21 log cycle reduction in surviving probiotic bacteria at the end of the 10-h exposure period. As demonstrated previously by Lim et al. (2015), the presence of live *S. cerevisiae* cells (positive control) significantly enhanced the survival of *L. rhamnosus* HN001 by 3.30 log cycles during the acid stress assay, whilst yeast counts decreased by 0.31 Log CFU/mL (Additional file 1: Figure S1). The addition of various IYDs also significantly improved the survival of the LAB by 2.75–4.05 log cycles with respect to the negative control, although the efficacy of each inactivated yeast preparation differed. When OptiRed^®^ was added, the probiotic cell count declined continuously throughout the incubation period, such that it was significantly lower than the negative control for the first 6 h to reach a final cell count of 6.94 Log CFU/mL (1.46 Log cycle reduction). The overall reduction in viable *L. rhamnosus* HN001 counts when OptiWhite^®^ and Noblesse^®^ were added were 0.25 and 0.15 Log CFU/mL, respectively, indicating that these two IYD had greater protective effects on the LAB than live yeast.

### Protective effects of water-soluble and insoluble components of inactivated yeast derivatives

**Fig. 2:**
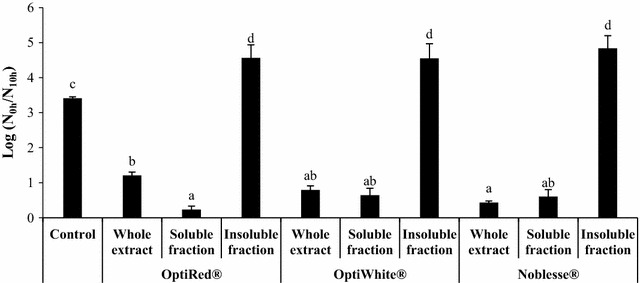
Effect of water-soluble and insoluble fractions of IYDs on the viability of *L. rhamnosus* HN001. Different lowercase letters indicate significant differences (*P* < 0.05). Values are the mean of triplicate experiments (*n* = 3), with *error bars* representing the standard deviations of the mean values

Considering the partial solubility of the three IYDs in the buffer used for the acid stress assay, the autoclaved IYD extracts were centrifuged to obtain the water-soluble (supernatant) and insoluble (pellet) fractions for preliminary investigation of the components responsible for enhancing the survival of *L. rhamnosus* HN001 in the acidic buffer. Figure 2 shows the changes in viable probiotic count after 10-h exposure of *L. rhamnosus* HN001 cells to pH 3.0 with the water-soluble and insoluble fractions of the three IYDs. Unexpectedly, an inhibitory effect was observed when all water-insoluble fractions were assayed as the survival of *L. rhamnosus* HN001 was significantly lower than the control by more than 1 log cycle. In contrast, the supernatants of the IYD extracts increased the survival of the probiotic LAB by 3.21, 2.80 and 2.88 log cycles for OptiRed^®^, OptiWhite^®^ and Noblesse^®^, respectively.

### Probiotic viability enhancing effects of water-soluble components of inactivated yeast derivatives after ultrafiltration

**Fig. 3:**
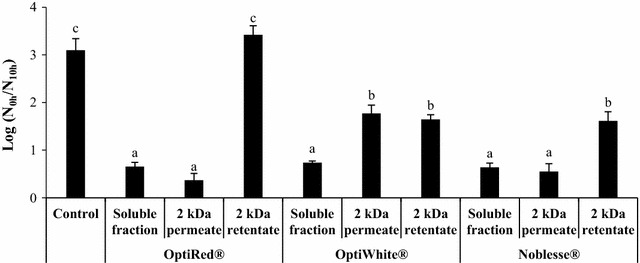
Effect of 2 kDa membrane permeates and retentates of IYD supernatants on *L. rhamnosus* HN001 viability. Different lowercase letters indicate significant differences (*P* < 0.05). Values are the mean of triplicate experiments (*n* = 3), with *error bars* representing the standard deviations of the mean values

Ultrafiltration was performed on the supernatants of the three IYD extracts for further examination of the water-soluble compounds involved in protecting *L. rhamnosus* HN001 from acid stress. A 2 kDa MWCO membrane was used to fractionate the supernatant components by molecular weight and the probiotic viability enhancing effect of permeates and retentates obtained is presented in Fig. 3. No protective effects were observed for the retentate of OptiRed^®^ supernatant, as the reduction in *L. rhamnosus* HN001 cell count (3.42 log cycles) was not significantly different from the control. On the other hand, the 2 kDa permeate of the OptiRed^®^ supernatant enhanced the survival of the LAB by 2.65 log cycles as the viable probiotic count declined by only 0.37 log cycles. This implies that the viability enhancing compounds present in the OptiRed^®^ were of low molecular weight, since the survival of *L. rhamnosus* HN001 at pH 3.0 was similar when the whole supernatant or it’s permeate was added. Both the 2 kDa permeate and retentate of the OptiWhite^®^ supernatant exerted similar protective effects on *L. rhamnosus* HN001, as the survival of the LAB was improved by 1.23 and 1.39 log cycles, respectively. Nevertheless, the OptiWhite^®^ supernatant was able to significantly enhance the viability of the probiotic to a greater extent than its ultrafiltered constituents, suggesting that a combination of low and high molecular weight compounds present in the IYD increased the acid resistance of the LAB. Similarly, the viability of *L. rhamnosus* HN001 at pH 3.0 was enhanced by both ultrafiltration fractions of the Noblesse^®^ supernatant. The survival of the probiotic was improved by 2.43 and 1.28 log cycles by the permeate and retentate, respectively, with the former conferring a significantly stronger protective effect on the LAB.

**Fig. 4:**
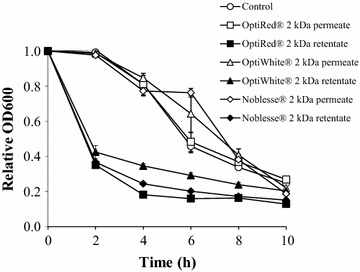
Aggregation of *L. rhamnosus* HN001 with 2 kDa membrane permeates and retentates of IYD supernatants. Values are the mean of triplicate experiments (*n* = 3), with *error bars* representing the standard deviations of the mean values

The influence of permeates and retentates on the aggregation of *L. rhamnosus* HN001 was evaluated to ascertain the role of bacterial aggregation as a possible mechanism of survival enhancement. It was observed that the 2 kDa permeates of all IYD supernatants did not affect the aggregation of *L. rhamnosus* HN001 in the pH 3.0 buffer, despite the improved survival of the LAB with these fractions (Fig. 4). Conversely, assays with the 2 kDa retentate of IYD supernatants had greater bacterial aggregation activity. The turbidity of probiotic cell suspensions containing retentates of OptiRed^®^, OptiWhite^®^ and Noblesse^®^ supernatants declined sharply within the first 2 h to 35, 42 and 37% of their initial value, respectively, whereas no significant changes were observed in assays with their corresponding permeates and the control.

### Chemical characterization of water-soluble fractions of inactivated yeast derivatives extracts

Chemical characterization of 2 kDa permeates and retentates obtained from the ultrafiltration of the three IYD supernatants was performed so as to provide insights into the components responsible for improving the survival of *L. rhamnosus* HN001 at pH 3.0.

Free amino acids and ammonia were detected in all 2 kDa permeates of the IYD supernatants, whereas none were present in the retentates as expected due to their low molecular weight. As shown in Table 1, it was noted that the three IYDs had quantitative and qualitative differences in their free amino acid composition. The total free amino acid concentration was the highest in the Noblesse^®^ permeate (162.07 μg/mL), followed by OptiRed^®^ (135.28 μg/mL) and the lowest in OptiWhite^®^ (109.43 μg/mL). For individual amino acids and ammonia, no histidine and tryptophan were detected in any of the IYDs, whilst cysteine and methionine were only found in OptiWhite^®^ and OptiRed^®^ permeates, respectively. Ammonia, glutamic acid, arginine, alanine and proline concentrations were the highest in the Noblesse^®^ permeate, whereas the OptiRed^®^ permeate had the greatest amounts of the remaining amino acids (aspartic acid, serine, glycine, threonine, tyrosine, valine, lysine, isoleucine, leucine and phenylalanine).

**Table 1:** Changes in free amino acid and ammonia concentrations of the 2 kDa membrane permeates of IYD supernatants

	Concentration at 0 h (μg/mL)			Concentration after 10 h (μg/mL)		
	OptiRed^®^	OptiWhite^®^	Noblesse^®^	OptiRed^®^	OptiWhite^®^	Noblesse^®^
Ammonia	1.99 ± 0.37^a^	3.48 ± 0.28^b^	6.89 ± 0.33^c^	2.32 ± 0.48^A^	3.61 ± 0.31^B^	6.09 ± 0.47^C^
Aspartic acid	17.06 ± 2.33^b^	4.38 ± 0.59^a^	5.41 ± 0.63^a^	16.30 ± 2.12^B^	4.54 ± 0.57^A^	4.41 ± 0.38^A^
Serine	8.43 ± 0.83^b^	4.68 ± 0.38^a^	5.98 ± 0.32^a^	7.74 ± 0.92^B^	4.75 ± 0.41^A^	4.71 ± 0.29^A*^
Glutamic acid	20.91 ± 1.43^a^	36.69 ± 1.41^b^	75.64 ± 2.29^c^	22.07 ± 2.99^A^	34.01 ± 3.20^B^	54.41 ± 2.70^C*^
Glycine	4.59 ± 0.06^b^	1.94 ± 0.25^a^	1.77 ± 0.13^a^	5.14 ± 1.00^B^	2.58 ± 0.42^A^	1.96 ± 0.30^A^
Histidine	ND	ND	ND	ND	ND	ND
Arginine	1.10 ± 0.18^a^	4.07 ± 0.54^b^	5.96 ± 0.19^c^	1.53 ± 0.19^A^	3.44 ± 1.05^B^	3.96 ± 0.70^B*^
Threonine	4.16 ± 0.07^c^	2.10 ± 0.20^b^	1.05 ± 0.07^a^	4.31 ± 0.59^C^	2.08 ± 0.62^B^	0.90 ± 0.14^A^
Alanine	21.37 ± 1.64^a^	22.32 ± 0.97^a^	30.83 ± 0.81^b^	18.85 ± 2.16^A^	20.70 ± 1.55^A^	21.97 ± 1.27^A*^
Proline	4.79 ± 0.26^a^	15.50 ± 1.87^b^	18.88 ± 0.53^c^	4.64 ± 0.77^A^	13.71 ± 1.22^B*^	13.70 ± 0.83^B*^
Cysteine	ND	0.94 ± 0.04	ND	ND	0.95 ± 0.14	ND
Tyrosine	5.31 ± 0.07^c^	1.66 ± 0.07^b^	0.79 ± 0.06^a^	4.97 ± 0.91^C^	1.73 ± 0.23^B^	0.61 ± 0.10^A^
Valine	10.85 ± 0.31^b^	5.11 ± 0.22^a^	4.60 ± 0.09^a^	10.53 ± 1.65^B^	5.14 ± 0.52^A^	3.60 ± 0.28^A*^
Methionine	2.08 ± 0.08	ND	ND	1.94 ± 0.36	ND	ND
Lysine	5.83 ± 0.70^b^	1.38 ± 0.15^a^	0.40 ± 0.05^a^	4.98 ± 0.86^B^	1.03 ± 0.14^A^	ND^*^
Isoleucine	7.39 ± 0.16^b^	1.58 ± 0.11^a^	1.56 ± 0.09^a^	7.23 ± 1.09^B^	1.83 ± 0.24^A^	1.25 ± 0.15^A*^
Leucine	11.94 ± 0.29^c^	2.87 ± 0.16^b^	1.83 ± 0.08^a^	11.46 ± 1.58^B^	3.18 ± 0.38^A^	1.57 ± 0.16^A^
Phenylalanine	7.49 ± 0.26^c^	1.65 ± 0.04^b^	0.80 ± 0.03^a^	7.25 ± 1.25^B^	1.93 ± 0.24^A^	0.76 ± 0.11^A^
Tryptophan	ND	ND	ND	ND	ND	ND
Total	135.28 ± 7.52^b^	109.43 ± 5.31^a^	162.07 ± 4.68^c^	131.25 ± 16.84^B^	104.21 ± 10.32^A^	119.44 ± 7.16^AB^*

Values are expressed as the mean ± standard deviation of three independent experiments (*n* = 3). Mean values in the same row with different lower and upper case letters are significantly different (*P* < 0.05) for 0 h and 10 h, respectively. Mean values denoted with * indicate significant difference (*P* < 0.05) between concentrations at 0 h and 10 h for each IYD

*ND* not detected

The concentrations of free amino acids and ammonia remaining after the 10-h acid stress assay are also presented in Table 1. For OptiRed^®^ permeate assays, there were no significant differences between the initial and final concentrations of all free amino acids tested and ammonia. Likewise for OptiWhite^®^ permeate assays, no net changes were observed, with the exception of a slight decrease in proline content. The utilization of amino acids by the LAB was more evident in Noblesse^®^ permeate assays, as significant reductions in serine, glutamic acid, arginine, alanine, proline, valine, lysine and isoleucine were observed after the incubation period. The difference in free amino acid utilization by *L. rhamnosus* HN001 amongst the three IYD permeates may be an indication of preferable substrates being present in OptiRed^®^ and OptiWhite^®^ as compared to Noblesse^®^.

**Fig. 5:**
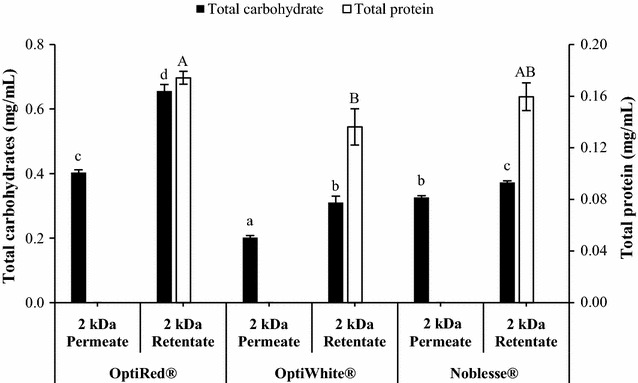
Total carbohydrate and protein concentrations of 2 kDa membrane permeates and retentates of IYD supernatants. Different lowercase and uppercase letters indicate significant differences (*P* < 0.05) for total carbohydrates and proteins, respectively. Values are the mean of triplicate experiments (*n* = 3), with *error bars* representing the standard deviations of the mean values

The total carbohydrate and protein concentrations of each ultrafiltered fraction of the IYD supernatants are shown in Fig. 5. For the water-soluble carbohydrates, significantly higher levels were present in the 2 kDa retentates as compared to their corresponding 2 kDa permeates. It was noted that OptiRed^®^ had the greatest amount of carbohydrates amongst the IYDs for both permeates and retentates, followed by Noblesse^®^ and the least in OptiWhite^®^. Analysis of free sugars by liquid chromatography revealed that no mono- and disaccharides were detected in all fractions, indicating that the water-soluble carbohydrates present in the IYDs were oligosaccharides and/or high-molecular weight polysaccharides in nature. Quantification of water-soluble proteins using the Coomassie brilliant blue G-250 dye-binding method showed that proteins were only found in the 2 kDa retentates of all the IYD supernatants and absent in permeates. Similar to the water-soluble carbohydrates, the protein content was the highest in the OptiRed^®^ retentate (0.17 mg/mL) and the lowest in the OptiWhite^®^ retentate (0.14 mg/mL).

**Fig. 6:**
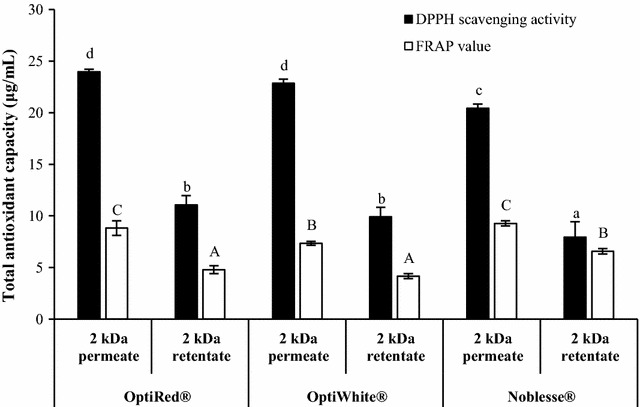
Total antioxidant capacities of 2 kDa membrane permeates and retentates of IYD supernatants. Different lowercase and uppercase letters indicate significant differences (*P* < 0.05) for DPPH scavenging activity and FRAP values, respectively. Values are the mean of triplicate experiments (*n* = 3), with *error bars* representing the standard deviations of the mean values

Figure 6 shows the total antioxidant activity of the six fractions measured using the DPPH and FRAP assays. In general, the antioxidant activities of the 2 kDa permeates were significantly higher than their respective retentates. The DPPH radical scavenging activity of permeates was at least two-fold greater than that of their corresponding retentates, with the OptiRed^®^ and OptiWhite^®^ fractions having significantly higher antioxidant activity than the respective Noblesse^®^ fractions. On the other hand, FRAP values of the OptiRed^®^ and Noblesse^®^ permeates were significantly higher than that of the OptiWhite^®^ permeate, whilst the FRAP value of the Noblesse^®^ retentate was greater than the other two retentates. Although OptiWhite^®^ is used to prevent browning in white wines due to its high glutathione content, the total antioxidant activity of its water-soluble components was not significantly different from OptiRed^®^. This was likely due to the degradation of glutathione during the autoclaving process which diminished the antioxidant property of OptiWhite^®^.

## Discussion

The present study served as a follow-up to previous studies involving the *L. rhamnosus* HN001 and *S. cerevisiae* EC-1118 pairing (Suharja et al. 2014; Lim et al. 2015). These studies demonstrated the potential of using viable and non-viable yeast as a means to enhance the acid tolerance of *L. rhamnosus* HN001. In the current study, we used three IYDs as sources of non-viable yeast and compared its efficacy against live *S. cerevisiae* EC-1118. We proposed several reasons for IYDs viability enhancing properties with reference to established mechanisms, based on results involving other strains of probiotics and lactic acid bacteria.

An acid stress assay that was modified from Lim et al. 2015 was used to methodically evaluate the viability enhancing properties of the IYD. Lactic acid was used to acidify the assay medium to pH 3.0 as it is the primary metabolite produced by *L. rhamnosus* during fermentation, as well as being an acid stressor responsible for lowering probiotic viability during the storage of fermented products (Gonçalves et al. 1997). In addition, lactic acid has a stronger bacteria inhibitory activity compared to the commonly used hydrochloric acid at the same pH value due to its higher acid dissociation constant, ability to modify cell surface properties and to induce cell membrane damage (Wang et al. 2014). An initial assessment of the IYDs probiotic viability enhancing property at a concentration of 3 g/L was performed in parallel with non-supplemented pH 3.0 buffer as a negative control and in the presence of viable *S. cerevisiae* EC-1118 cells as a positive control. The results showed that OptiWhite^®^ and Noblesse^®^ improved the survival of the LAB to a greater extent than the live yeast, which itself has been demonstrated to enhance acid resistance of *L. rhamnosus* HN001 in both acidic buffer and fermented milk systems in previous studies (Lim et al. 2015; Suharja et al. 2014). It is also worthwhile to note that the IYD extracts were sterilized by autoclaving, indicating that the viability enhancing components present were either thermal stable or that adequate amounts were retained after the heat treatment. Similar stability was also observed by Andujar-Ortiz et al. (2010), whereby aqueous extracts of IYDs obtained through pressurized liquid extraction under a harsher temperature condition exhibited a growth-stimulating effect on wine LAB.

Subsequent acid stress assays with supernatants and pellets of the autoclaved IYD extracts provided a clearer delineation of the nature of the protective components, given that the water-soluble and insoluble fractions of all three IYDs exerted opposite effects on the viability of *L. rhamnosus* HN001. Besides water-insoluble polysaccharides (inert cellulose supports and high molecular weight β-glucans), the pellets of the IYD extracts could comprise of compounds with lower polarity including fatty acids, long-chain carboxylic esters and heterocyclic nitrogenous compounds which may have inhibitory activities against the LAB by disrupting membrane integrity and cellular functions (Andujar-Ortiz et al. 2010; Ángeles Pozo-Bayón et al. 2009; Bonde and Gaikwad 2004). In contrast, supernatants enhanced the viability of the probiotic bacteria, denoting that the compounds in IYDs that enhanced the acid resistance of *L. rhamnosus* HN001 during acid stress were water-soluble.

Fractionation of the supernatants by ultrafiltration separated the water-soluble components in the three IYD extracts by their molecular weights. Based on the results of acid stress assays with 2 kDa MWCO permeates and retentates, low molecular weight (<2 kDa) compounds present in OptiRed^®^, OptiWhite^®^ and Noblesse^®^ protected *L. rhamnosus* HN001 from acid stress, with higher molecular weight (>2 kDa) compounds in the latter two IYDs also improving the survival of the LAB. Several studies have reported enhanced acid resistance in probiotic bacteria by yeasts resulting from the co-aggregation between the two groups of microorganisms (Lim et al. 2015; Xie et al. 2012). Similar aggregation kinetics between the control and assays with permeates excluded the induction of bacterial aggregation as the protective mechanism of these fractions. Despite increasing aggregation of *L. rhamnosus* HN001, the ultrafiltered OptiRed^®^ supernatant retentate offered no protective effect which was unexpected. On the other hand, aggregation-inducing activity of retentates obtained from the OptiWhite^®^ and Noblesse^®^ supernatants may have contributed to their probiotic viability-enhancing effect. To identify other possible survival enhancing mechanisms of the IYDs, chemical analysis of carbohydrates, amino acids, proteins and antioxidant activity of the permeates and retentates were carried out.

Amongst the various acid tolerance responses in LAB, the expulsion of protons from the cell through F_0_F_1_-ATPase complexes found on the bacterial membrane is the most well understood mechanism. This process is energetically demanding as the translocation of protons out of the bacterial cytoplasm by F_0_F_1_-ATPase is coupled to ATP hydrolysis (Siegumfeldt et al. 2000). In LAB, the ATP required is mainly generated from the glycolysis of sugars via the Embden–Meyerhof pathway or pentose phosphate pathway (Corcoran et al. 2005). Although carbohydrates were present in both 2 kDa permeates or retentates of the IYD supernatants, no free mono- and disaccharides were detected. Thus, *L. rhamnosus* HN001 might have utilized complex carbohydrates as a source of metabolizable sugars through hydrolysis with glycosidases (Kankainen et al. 2009). Prior research involving IYDs used in the current study and similar preparations had characterized the carbohydrates in these preparations as yeast cell wall polysaccharides composed of mannose and/or glucose monomers (Andujar-Ortiz et al. 2010; Perez-Magarino et al. 2015). Based on the pore size of the membrane used for ultrafiltration (2 kDa MWCO), the carbohydrates in permeates were probably oligosaccharides and low molecular weight polysaccharides such as mannooligosaccharides and water-soluble glucans, while those in the retentates had higher degrees of polymerization and branching.

High molecular weight mannans likely accounted for part of the carbohydrates quantified in retentates, considering that the IYDs in this study are used for increasing the content of parietal yeast mannoproteins in wines. The detection of proteins and results of aggregation assays with the 2 kDa retentates also affirmed the presence of these glycoproteins, since lectin-like proteins on the cell surface of LAB can bind to mannose residues of yeast mannans and mannoproteins, causing an increase in bacterial aggregation (Katakura et al. 2010). Size exclusion chromatography of ethanol-precipitated polysaccharides in OptiRed^®^ and Noblesse^®^ performed by Gonzalez-Royo et al. (2013) revealed that the former IYD comprised of polysaccharides that were greater than 5 kDa, whilst the latter had polysaccharides that were mostly smaller than 6 kDa. The variations in polysaccharide structure amongst the IYDs may have contributed to the differences in their probiotic viability enhancing effect since the hydrolysis of carbohydrate polymers by glycosidases can be affected by their molecular weight, glycosidic linkages and extent of branching (Sarbini et al. 2011).

The presence of free amino acids in the 2 kDa permeates, particularly for Noblesse^®^, could also have improved the acid resistance of *L. rhamnosus* HN001 through the homeostatic regulation of cytoplasmic pH via decarboxylation, deimination and branched-chain amino acid (BCAA) catabolism reactions. Decarboxylation of glutamic acid to γ-aminobutyric acid by the LAB results in the release of carbon dioxide and increases intracellular pH by assimilating a proton (Siragusa et al. 2007). The arginine deiminase pathway in LAB raises the cytoplasmic pH by producing two moles of ammonia per mole of arginine metabolized. Additionally, the catabolism of arginine generates ATP which can be utilized for F_0_F_1_-ATPase activity (Liu et al. 2003; Rollan et al. 2003). Under conditions of acid stress and carbohydrate starvation, LAB can switch from sugar to amino acid catabolism for energy production, as ATP is formed during the biosynthesis of branched-chain fatty acids from BCAAs (Serrazanetti et al. 2011). Glutamate is also produced via transamination of amino acids with α-ketoglutarate during the initiation of BCAA catabolism, further contributing to the regulation of intracellular pH as mentioned above (Williams et al. 2004).

Although free amino acids were absent in the 2 kDa retentates, the proteins present may have been utilized by *L. rhamnosus* HN001 for the production of amino acids (Aljewicz et al. 2014). These soluble proteins includes peptides produced during yeast autolysis or the protein moiety of mannoproteins, which could be hydrolyzed by proteases and oligopeptidases in the LAB (Koponen et al. 2012; Pozo-Bayon et al. 2009). Exposure of LAB to adverse environmental conditions of heat, acidic and osmotic stress induces an upregulation of protein expression as a response mechanism (van de Guchte et al. 2002). In the acid stress assay, supplementing IYDs also provides a source of amino acids required for the synthesis of general stress response (ClpE, DnaK, GrpE) and cell signalling (LuxS) proteins, enzymes involved in carbohydrate, protein and amino acid metabolism, transporter molecules and F_0_F_1_-ATPase (Koponen et al. 2012).

Apart from lowering cytoplasmic pH, lactic acid has also been shown to exert oxidative stress in LAB by dissociating iron from the catalytic sites of proteins and forming iron-lactate complexes. The increase in free iron and iron-lactate complexes enhances the production of highly reactive hydroxyl radicals from hydrogen peroxide by catalysing the Fenton reaction (Bruno-Bárcena et al. 2010). Antioxidants in IYDs might have contributed to the improved viability of *L. rhamnosus* HN001 in the acid stress assays by alleviating oxidative stress imposed by reactive oxygen species. The greater survival of the LAB at pH 3.0 with IYD supernatant permeates as compared to retentates may be partly due to their higher antioxidant activity. Compounds that could contribute to the antioxidant activity of IYDs include aromatic amino acids, peptides and low molecular weight, water-soluble glucans (Alcaide-Hidalgo et al. 2007; Lei et al. 2015; Nimalaratne et al. 2011).

In conclusion, this study demonstrated that polysaccharide-rich IYDs used in winemaking improved the survival of the probiotic bacteria *L. rhamnosus* HN001 in a pH 3.0 buffer system. The protective property of these preparations was attributed to their water-soluble components, which varied amongst the different IYDs. Nonetheless, further characterization of carbohydrates and proteins is required to account for the differences in the viability enhancing effect of the three IYDs higher molecular weight fractions. The IYDs should also be screened on other probiotic bacteria strains to assess the robustness of their protective effects. Lastly, it would be prudent to evaluate the effectiveness of IYDs in prolonging the viability of probiotics in food matrices to account for the interactions between various components and the impact of the yeasts preparations on the organoleptic properties of the food, which is currently underway.

## Additional file

**Additional file 1: Figure S1.** Viability of *S. cerevisiae* EC-1118 when co-incubated with *L. rhamnosus* HN001 at pH 3.0. Cell counts are the mean values of triplicate experiments (n = 3), with error bars representing the standard deviation of the mean values.

## Abbreviations

IYD: inactivated yeast derivative
LAB: lactic acid bacteria
MRS: de Man, Rogosa, Sharpe
YM: yeast-malt
PBS: phosphate-buffered saline
RC: regenerated cellulose
MWCO: molecular weight cut off
UFLC: ultra-fast liquid chromatography
AQC: aminoquinolyl-*N*-hydroxysuccinimidyl carbamate
DPPH: 1,1-diphenyl-2-picrylhydrazyl
FRAP: ferric reducing ability of plasma
